# Modeling the tumor microenvironment of anaplastic thyroid cancer: an orthotopic tumor model in C57BL/6 mice

**DOI:** 10.3389/fimmu.2023.1187388

**Published:** 2023-07-21

**Authors:** Zhen Xu, Hyo Shik Shin, Yoo Hyung Kim, Seong Yun Ha, Jae-Kyung Won, Su-jin Kim, Young Joo Park, Sareh Parangi, Sun Wook Cho, Kyu Eun Lee

**Affiliations:** ^1^ Cancer Research Institute, Seoul National University College of Medicine, Seoul, Republic of Korea; ^2^ Department of Surgery, YanBian University Hospital, Yanji, Jilin, China; ^3^ Department of Internal Medicine, Seoul National University College of Medicine, Seoul, Republic of Korea; ^4^ Department of Internal Medicine, Seoul National University Hospital, Seoul, Republic of Korea; ^5^ Department of Pathology, Seoul National University Hospital, Seoul, Republic of Korea; ^6^ Department of Pathology, College of Medicine, Seoul National University, Seoul, Republic of Korea; ^7^ Department of Surgery, Seoul National University College of Medicine, Seoul, Republic of Korea; ^8^ Division of Surgery, Thyroid Center, Seoul National University Cancer Hospital, Seoul, Republic of Korea; ^9^ Department of Molecular Medicine and Biopharmaceutical Sciences Graduate School of Convergence Science and Technology, Seoul National University, Seoul, Republic of Korea; ^10^ Department of Surgery, Massachusetts General Hospital, Harvard Medical School, Boston, MA, United States

**Keywords:** anaplastic thyroid cancer, syngeneic, tumor microenvironment, immunotherapy, BRAF V600E

## Abstract

**Introduction:**

Securing a well-established mouse model is important in identifying and validating new therapeutic targets for immuno-oncology. The C57BL/6 mouse is one of the most fully characterised immune system of any animal and provides powerful platform for immuno-oncology discovery. An orthotopic tumor model has been established using TBP3743 (murine anaplastic thyroid cancer [ATC]) cells in B6129SF1 hybrid mice, this model has limited data on tumor immunology than C57BL/6 inbred mice. This study aimed to establish a novel orthotopic ATC model in C57BL/6 mice and characterize the tumor microenvironment focusing immunity in the model.

**Methods:**

Adapted TBP3743 cells were generated via *in vivo* serial passaging in C57BL/6 mice. Subsequently, the following orthotopic tumor models were established via intrathyroidal injection: B6129SF1 mice injected with original TBP3743 cells (original/129), B6129SF1 mice injected with adapted cells (adapted/129), and C57BL/6 mice injected with adapted cells (adapted/B6).

**Results:**

The adapted TBP3743 cells de-differentiated but exhibited cell morphology, viability, and migration/invasion potential comparable with those of original cells *in vitro*. The adapted/129 contained a higher Ki-67^+^ cell fraction than the original/129. RNA sequencing data of orthotopic tumors revealed enhanced oncogenic properties in the adapted/129 compared with those in the original/129. In contrast, the orthotopic tumors grown in the adapted/B6 were smaller, with a lower Ki-67^+^ cell fraction than those in the adapted/129. However, the oncogenic properties of the tumors within the adapted/B6 and adapted/129 were similar. Immune-related pathways were enriched in the adapted/B6 compared with those in the adapted/129. Flow cytometric analysis of the orthotopic tumors revealed higher cytotoxic CD8^+^ T cell and monocytic-myeloid-derived suppressor cell fractions in the adapted/B6 compared with the adapted/129. The estimated CD8^+^ and CD4^+^ cell fractions in the adapted/B6 were similar to those in human ATCs but negligible in the original/B6.

**Conclusion:**

A novel orthotopic tumor model of ATC was established in C57BL/6 mice. Compared with the original B6129SF1 murine model, the novel model exhibited more aggressive tumor cell behaviours and strong immune responses. We expect that this novel model contributes to the understanding tumor microenvironment and provides the platform for drug development.

## Introduction

Anaplastic thyroid carcinoma (ATC) is a highly aggressive malignant tumor accounting for 1–2% of all thyroid cancers (1–5). Upon initial presentation, most patients exhibit extensive local invasion or distant metastatic lesions (stage IVA-IVC) (2). Thus, the associated prognosis is poor, with a median overall survival of 5–12 months (2, 4, 6).

Tremendous research efforts have sought to characterize the genetic and molecular pathogenesis of ATC (4, 7–9), leading to the development of novel targeted therapies. For instance, the BRAF^V600E^ mutation is frequently found in ATCs (20–50% of the cases) (7, 8, 10). The combination therapy of BRAF and MEK inhibitors has demonstrated therapeutic efficacy in BRAF^V600E^-mutated ATCs (11), leading to its approval in 2018 by the United States Food and Drug Administration. However, progression-free and median overall survival remain relatively low (6.7 and 14.5 months, respectively) even after the combination therapy (12).

One of requirements for the development of anti-cancer drugs is the establishment of a mouse model that replicates the histology and microenvironment of human tumors (13). The cultivated cell-line derived xenograft model (CDXM) is currently the most widely used model for drug screening and development (14). Although the CDXM exhibits rapid tumor development and excellent experimental reproducibility, it is unsuitable for investigating tumor microenvironments as it is established in immune-compromised mice. Meanwhile, the genetically engineered model (GEM) has been successfully applied to replicate and analyse tumor microenvironments. However, tumor formation requires a longer period in the GEM than in the xenograft model, and the rate of tumor formation varies among mice (15–17). Thus, by combining the advantages of the CDXM and GEM, a model for transplanting murine tumor cells from the GEM back into mice has been developed. The TBP3743 murine ATC cell line generated from mice with a thyroid-specific *Braf* ^V600E^ mutation and *Trp53* deletion (18, 19) exhibits rapid and consistent tumor growth following orthotopic injection in B6129SF1 hybrid mice (19). Although this model has been tried to explore the tumor microenvironment and assess the anti-tumor efficacy of immune checkpoint inhibitors (20), B6129SF1 hybrid mice have little cumulative data regarding tumor biology and drug development compared to inbred C57BL/6 mice (21).

In this study, we performed *in vivo* adaptation of TBP3743 cells within inbred C57BL/6 mice and established an orthotopic model of ATC. We characterized the differentiation status, oncogenic molecular features, and immune signatures of the tumors from the original and the adapted TBP3743 cells. The findings of this study describe a suitable preclinical mouse model for investigating the ATC tumor microenvironment and developing anti-cancer drugs for immunotherapy.

## Methods

### Maintenance of tumor cells *in vitro*

The original TBP3743 cell line (19), generated from B6129SF1 mice with thyroid-specific *Braf^V600E^* and thyroid-specific *Trp53* deletion, was kindly gifted from Dr. Sareh Parangi (Department of Surgery, Massachusetts General Hospital, Harvard Medical School, Boston, MA, USA). These cells were maintained in RPMI-1640 media supplemented with 10% fetal bovine serum (FBS; WELGENE, Seoul, Korea), 2 mM GlutaMAX™ (Gibco, New York, NY, USA), and 100 U/mL penicillin-streptomycin (Gibco).

### Generation of adapted TBP3743 cells in C57BL/6 mice

Six-week-old female C57BL/6 and B6129SF1 mice were purchased from Orient Bio, Inc. (Seongnam, Korea) and the Jackson Laboratory (Bar Harbor, ME, USA), respectively. A mixture of 1 × 10^7^ original TBP3743 cells and Matrigel (BD Biosciences, Franklin Lakes, NJ) was injected subcutaneously into C57BL/6 mice (*n* = 5) for generating adapted TBP3743 cells. Two-dimensional tumor size was measured twice per week, and the tumor volume was calculated using Eq. (1):


(1) Volume = 12 × a × b2

where a is the longest diameter, and b is the perpendicular diameter (22). First, the mice were euthanized and the largest tumor was selected, surgically removed, minced, and incubated with phosphate-buffered saline (PBS) containing 2 mg/mL collagenase type 1 on a shaker at 37°C for 3 h. The cells were then maintained in RPMI-1640 media supplemented with 10% FBS. Expanded tumor cells were re-implanted in new C57BL/6 mice (*n* = 5) in the same manner as described above.

### Establishment of the murine orthotopic tumor model of ATC

The orthotopic tumor model was established by intrathyroidal injection of tumor cells. For intrathyroidal injection, 1 × 10^5^ tumor cells in 10 µL of PBS were injected into the left thyroid gland of mice using a Hamilton syringe with a 30G needle.

Six-week-old female C57BL/6 or B6129SF1 mice underwent intrathyroidal injection of the original or adapted TBP3743 cells. The original cells were injected into the B6129SF1 mice (original/129 group). Adapted cells after 3^rd^ and 6^th^ adaptation passage were injected into the B6129SF1 mice (adapted/129 group) or C57BL/6 (adapted/B6 group and SC6/B6 group, respectively). To evaluate the effect of tumor-host immune interactions in tumor growth, we injected the original and adapted cells into athymic mice (Orient Bio, Inc., Seongnam, Korea). Tumor size and volume were measured 9 days after tumor cell injection.

### Evaluation of the *in vivo* anti-tumor response to BRAF inhibitor in mice injected with adapted TBP3743 cells

To evaluate the anti-tumor response to a BRAF inhibitor, we labelled adapted TBP3743 cells with luciferase protein before generating the orthotopic thyroid cancer model. Beginning three days after tumor cell injection, mice were intraperitoneally injected daily with 10 mg/kg of PLX-4032 (Selleckchem, Houston, TX, USA; *n* = 8) or saline (untreated group; *n* = 8). To obtain *in vivo* bioluminescence imaging, mice were intraperitoneally injected with 100 µL (3 mg/mL PBS) of D-Luciferin (Perkin Elmer, Wellesley, MA). Bioluminescence imaging (IVIS spectrum, Perkin Elmer) was performed two weeks after tumor cell injection.

### Evaluation of the *in vivo* anti-tumor response to anti-PD-L1 antibody in mice injected with adapted TBP3743 cells

To evaluate the anti-tumor response to anti-PD-L1 antibody, we intraperitoneally injected daily with 0.2 mg/kg of anti-PD-L1 antibody (kindly gifted from Pf. Dae Hee Kim, Kangwon National University, Gangwon-do, Republic of Korea) or Ig G controls from 5 days after tumor cell injection. Tumor size and volume were measured 10 days after tumor cell injection.

### Immunohistochemical staining

The harvested tumors were fixed in 4% paraformaldehyde and embedded in paraffin. Immunohistochemistry (IHC) staining was performed with the following primary antibodies: anti-CD3 (1:100, ab5690, Abcam, Cambridge, UK), anti-CD4 (1:100, ab183685, Abcam), anti-CD8 (1:100, ab209775, Abcam), F4/80 (1:50, 14-4801-81, Invitrogen, San Diego, CA, USA), anti-CD163 (1:200, ab182422, Abcam), and anti-Ki67 (1:200, MA5-14520, Invitrogen).

### Cell viability assay

To compare the viability of original and adapted TBP3743 cells, 8 × 10^3^ cells/mL were seeded into a 96-well tissue culture plate with 100 µL of media. Subsequently, 10 µL of the CCK-8 solution (Dojindo, Kumamoto, Japan) was added to each well after 12, 36, and 60 h. The cells were incubated for an additional 2 h, and the absorbance was measured at 450 nm with a microplate reader (SpectraMax 190; Molecular Devices, San Jose, CA, USA).

To assess the drug response to the BRAF inhibitor, the original and adapted TBP3743 cells (1 × 10^3^/well) were seeded in 384-well plates and treated with PLX-4032 (0.05, 0.15, 0.46, 1.37, 4.12, 12.35, 37.03, 111.11, 333.33, or 1000 μM). Cell viability was evaluated using a CellTiter-Glo Luminescent Cell Viability Assay (Promega) after 72 h of PLX-4032 treatment.

### Real-time PCR assay

Total RNA was extracted using TRIzol reagent (Invitrogen) from *in vitro* maintained tumor cells or *in vivo* orthotopic tumors, and cDNA was synthesized using the M-MLV Reverse Transcriptase kit (Invitrogen). The real-time PCR was performed with the StepOne Plus real-time PCR system (Applied Biosystems) and TB green Premix (Takara Bio Inc., Otsu, Japan). The assay was performed according to the manufacturer’s protocol. Results were presented as the average of three independent experiments. Detailed primer sequences are listed in supplementary data (**Supplementary Table 1**).

### Cell migration assay

Cell migration was assessed using the Transwell system (Corning, NY, USA). Briefly, polycarbonate membranes with 8 µm pores were coated with 0.2% gelatin solution for 1 h and dried overnight. Cells were added to the upper chamber and placed into a 24-well plate. The lower chamber was filled with 650 µL of serum-free media. After 12 h, non-migrating cells were swabbed with a cotton-tipped applicator. Migrating cells to lower chamber were fixed in methanol for 30 min, and stained with 1% crystal violet for 30 min. The results were quantified with ImageJ.

### 3D invasion assay

A 3D invasion assay was performed with the original and adapted TBP3743 cells using a Cultrex 3D Spheroid Cell Invasion Assay (Trevigen, Inc., Gaithersburg, MD, USA). Briefly, 2 × 10^3^ cells were dispensed in 50 µL of Spheroid Formation Extracellular Matrix (ECM) per well and incubated at 37 °C for 72 h. After three days, 50 µL of the Invasion Matrix was added to each well and incubated at 37°C for seven days. Cell invasion was observed microscopically using the 4× objective, and spheroid images were analysed by ImageJ.

### Flow cytometry

Tumors were minced and incubated with Hanks’ Balanced Salt solution (HBSS) containing 2 mg/mL collagenase type 1 on a shaker at 37 °C for 3 h. Cells were washed and suspended in PBS supplemented with 2% FBS, followed by staining with fluorochrome-conjugated antibodies: anti-CD45 (1:250, 103138, BioLegend, San Diego, CA, USA), anti-CD3 (1:60, 35-0031-82, Invitrogen), anti-CD4 (1:60, 100422, BioLegend), anti-CD8 (1:60, 100714, BioLegend), anti-CD25 (1:10, MA5-17818, Invitrogen), anti-FoxP3 (1:10, 17-5773-82, Invitrogen), anti-CD11b (1:50, 101206, BioLegend), anti-Ly6C (1:50, 128026, BioLegend), anti-Ly6G (1:100, 560599, BD Biosciences, Franklin Lakes, NJ, USA), anti-F4/80 (1:50, 12-4801-82, Invitrogen), anti-CD80 (1:50, 562504, BD Biosciences), anti-CD206 (1:50, 46-2061-82, Invitrogen), anti-MHC Class 1 (1:50, ab95572, Abcam), and anti- NK1.1 (1:50, 61-5941-82, Invitrogen). Multicolour flow cytometry was performed using The MACSQuant® Analyzer 16 (Miltenyi Biotec B.V. & Co. KG, Bergisch Gladbach, Germany) and analysed with FlowJo software (BD Biosciences, OR, USA).

### Bulk RNA sequencing and data analysis

RNA was isolated from three tumor tissues in the original/129, adapted/129, and adapted/B6 groups. Total RNA was used to construct cDNA libraries with the TrueSeq RNA library kit (Illumina, San Diego, CA, USA) according to the manufacturer’s instructions. Next, sequencing was performed using an Illumina HiSeq2000 (Illumina) platform with ~100 nt paired-end reads. The quality of raw sequence data was assessed using FastQC (FastQC 0.11.3). Sequenced reads were aligned to the mm10 mouse genome assembly (GRCm38) reference genome with HISAT2 aligner (HISAT2 2.2.1). Raw read counts were used to analyse the differentially expressed genes (DEGs) among the groups by applying the Cuffdiff workflow (Cuffdiff 2.2.1). DEGs were defined as genes with a false discovery rate (FDR)< 0.05 and an absolute log2 fold change >1.

Curated gene sets were derived from the Kyoto Encyclopedia of Genes and Genomes (KEGG) pathway database and the Broad Institute Molecular Signatures Database. Over-representation analysis with the DEGs was performed using the DAVID functional annotation tool, and enriched gene sets were those with an FDR< 0.05. Single sample gene set enrichment analysis (ssGSEA) was performed with R package gene set variation analysis.

In the network analysis, nodes represented enriched gene sets that were significantly over-represented by DEGs, while edges represented gene expression profiles that were significantly correlated (similarity > 0.5) across samples/gene sets. Interpretation and plotting of the network were performed with R package igraph.

Immune cell proportions were analysed by CIBERSORT using the LM22 gene signature. Gene expression data of tumors from original/129, adapted/129, or adapted/B6, and human ATCs from the previously published data (8) were normalized as fragments per kilobase of transcript per million (FPKM) by Cuffnorm and uploaded to the CIBERSORT web portal (https://cibersort.stanford.edu/). Twenty-two human hematopoietic cell proportions per sample were provided.

### Statistical analysis

Statistical analyses were performed using GraphPad PRISM version 8.0.0 (GraphPad Software, San Diego, CA, USA). Data were presented as mean ± standard deviation. The independent *t*-test, paired *t*-test, or Mann–Whitney U-test (*n*< 10) was used to compare continuous variables. Statistical significance was defined as two-sided *P*-values< 0.05. Statistical analyses and plotting for bulk RNA sequencing were performed using R Statistical Software (version 4.0.3).

## Results

### Establishment of the adapted TBP3743 cell line via *in vivo* passaging to the C57BL/6 mice

**Figure 1:**
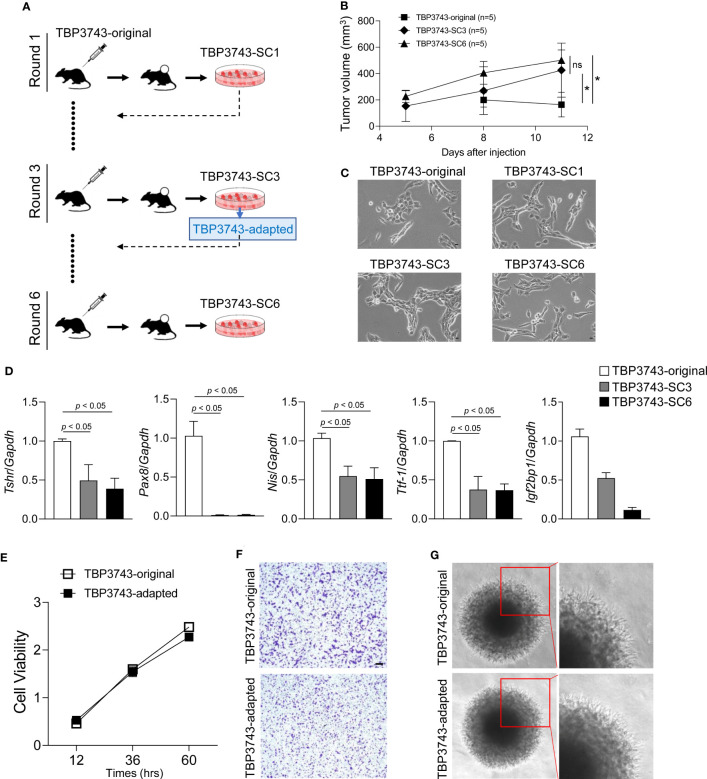
*In vivo* passaging of TBP3743 cells. TBP3743 cells were subcutaneously injected into C57BL/6 mice and tumor growth were observed for 21 days in 1st and 2nd round, and 10-15 days in 3rd, 4th 5th, and 6th round of *in vivo* passaging. At each round, the two largest tumors were harvested, primary single cell culture were performed, and established cells were used for the next round of *in vivo* passaging. **(A)** Scheme for TBP3743 adaptation into C57BL/6 mice. **(B)** Tumor growth curves of the original, 3rd and 6th round from day 5 to 11. **(C)** The morphology of TBP3743 original or subclones (scale bars: 200 µm). **(D)** Thyroid differentiation genes were analyzed by real-time RT-PCR. To characterize TBP3743-original or -adapted cells, **(E)** Cell proliferation were measured by the CCK-8 assay at the indicated time, **(F)** Cell migration assay were performed using Transwell system (scale bars: 50 µm), **(G)** Cell invasion ability were measured by spheroid culture system. SC, subclone.

To generate the adapted TBP3743 cells in C57BL/6 mice, six rounds of *in vivo* serial passaging were performed (**Figure 1A**). Cells harvested from round-1, -3, and -6 tumors were designated as SC1, SC3, and SC6 cells, respectively, and injected into another C57BL/6 mouse for the subsequent round. Tumors after rounds 3 were stably established three weeks after the injection (**Supplementary Table 2**). SC3 and SC6 exhibited comparable tumor growth *in vivo* (**Figure 1B**). The gross morphology of the original cells was similar to those of SC1, SC3, and SC6 cells (**Figure 1C**). The *Braf* ^V600E^ mutation was identified in all SC1–4 cells (**Supplementary Figure 1**). We compared the expression of genes associated with thyroid differentiation. The mRNA expressions of *Tshr*, *Pax8*, *Nis*, and *Ttf1* were significantly downregulated in SC3 and SC6 cells compared with the original TBP3743 cells, but comparable between SC3 and SC6 (**Figure 1D**). Consistently, protein expression of PAX8 was completely lost in SC3 and SC6, and that of TTF1 was lower, but still preserved in SC3 and SC6 compared with the original TBP3743 cells. TSHR protein expression was negligible in the original cell, SC3, and SC6 (**Supplementary Figure 2**). In addition, the expression of *Igf2bp1*, which has recently been identified as a marker for ATC (23), was observed to be significantly downregulated gradually from the original state to SC3 and further to SC6 (**Figure 1D**). In summary, the SC3 and SC6 cells were de-differentiated clones compared with the original cells. *In vivo* tumorigenic capacities between SC3 and SC6 in C57BL/6 mice were similar. *In vitro* cell morphology and the expressions of thyroid differentiation genes were all comparable between SC3 and SC6. Both SC3 and SC6 maintained the molecular characteristics of ATC. Thus, we designated SC3 as the adapted TBP3743 cells for further use.

Next, *in vitro* characteristics were investigated between the original and adapted TBP3743 cells. Cell viability was similar between two cell types (**Figure 1E**). The migration (**Figure 1F**, **Supplementary Figure 3**) and invasion (**Figure 1G**) capacity of these two cells were also comparable. Consistent with a previous report (19), loss of E-cadherin was observed in both cells. The abundances of N-cadherin and vimentin were similar (**Supplementary Figure 4A**). Regarding oncogenic signalling, the phosphorylation of AKT was significantly increased in the adapted TBP3743 cells compared to the original cells (**Supplementary Figure 4B**). Additionally, ERK phosphorylation was higher in the adapted TBP3743 cells compared to the original cells, but the difference did not reach statistical significance (**Supplementary Figure 4B**). Collectively, cell viability, migration, and invasion capacity were similar between the original and adapted cells, while the PI3K-AKT pathway showed a modest increase in the adapted TBP3743 cells compared with the original cells.

### Establishment of an orthotopic tumor model using adapted TBP3743 cells

**Figure 2:**
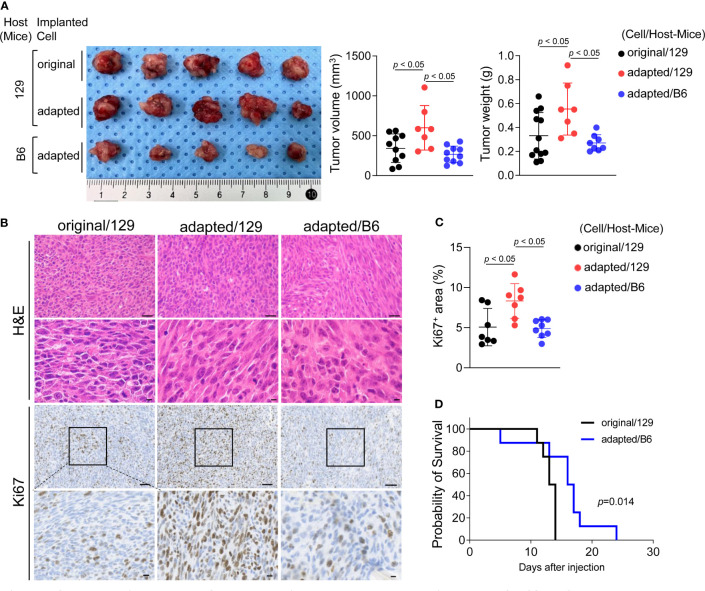
Establishment of orthotopic tumor model using TBP3743-B6 cells. B6129SF1 mice were implanted with 10^5^ cells of each cell line (TBP3743-original and -adapted cells) and C57BL/6 mice were implanted with 10^5^ cells of TBP3743-adapted. After 14 days post-implantation, tumors were harvested. **(A)** Representative image of tumors and, quantification of tumor volume and weight. **(B)** Representative image of H&E staining and Ki67 immunohistochemistry staining on each tumors (H&E upper panel scale bars: 50 µm; lower panel scale bars: 10 µm; Ki67 panel scale bar: 50 µm; lower panel scale bars: 10 µm). **(C)** Quantification of Ki67^+^ area in tumors. **(D)** FACS analysis of MHC class 1 on TBP3743-original, -adapted, and SC6 tumors. 129, B6129SF1; B6, C57BL/6. All data are expressed as mean ± SD.

Orthotopic tumor growth was investigated two weeks after tumor cell injection. The mean volume and weight of tumors collected from the adapted/129 group was greater than that of the original/129 group (volume: 343.8 ± 179.2 mm^3^ vs. 599.1 ± 278.9 mm^3^, *P<* 0.05; weight: 0.33 ± 0.19 g vs. 0.55 ± 0.22 g, *P<* 0.05, respectively; **Figure 2A**). The Ki-67^+^ cell fraction was markedly higher in the adapted/129 group than the original/129 group (**Figures 2B, C**). Histological features were similar among the groups, presenting with spindle sarcoma-like tumor cells with pleomorphic nuclei and high mitotic count, which are consistent with ATC features (**Figure 2B**). Hence, the adapted TBP3743 cells exhibited more proliferative characteristics than the original cells in the B6129SF1 mice. However, the tumor volume, weight, and Ki-67^+^ cell fraction from the adapted/B6 group were considerably lower than those from the adapted/129 group. Additionally, the median survival was significantly shorter in the original/129 group compared to those of the adapted/B6 group (median [range], 13.5 [11-14] vs 16.5 [5-24] days, P=0.014, **Figure 2D**)

### Molecular characteristics of adapted TBP3743 cell-derived orthotopic tumors

**Figure 3:**
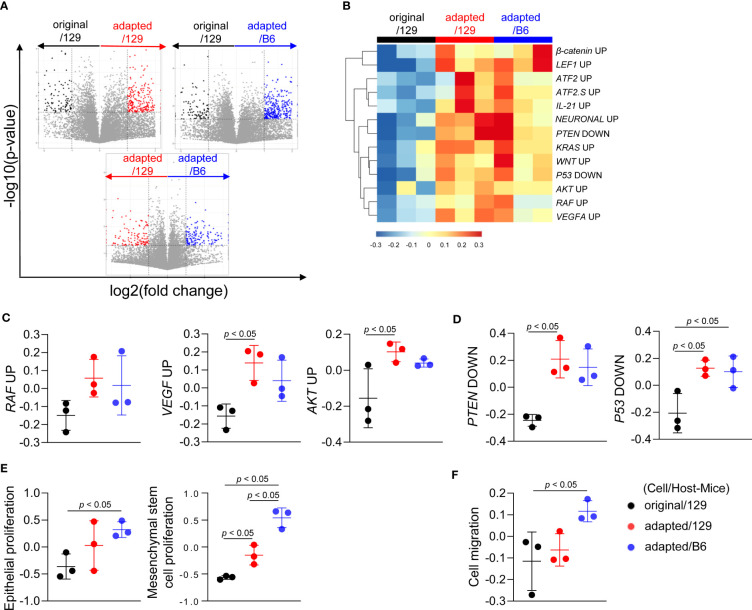
Molecular characteristics of orthotopic tumors from the adapted TBP3743 cells. B6129SF1 mice were implanted with 10^5^ cells of each cell line (TBP3743-original and -adapted cells) and C57BL/6 mice were implanted with 10^5^ cells of TBP3743-adapted. After 14 days post-implantation, tumors were harvested. Total RNA was harvested from whole tumor lysates and sequenced. **(A)** Volcano plots showing changes of gene expression. **(B)** Heatmap of changes in oncogenic signalling pathways and **(C, D)** each of oncogenic pathways. **(E)** Proliferation of epithelial and mesenchymal stem cells and **(F)** cell migration analysis. 129, B6129SF1; B6, C57BL/6. All data are expressed as mean ± SD.

To investigate the molecular characteristics of each orthotopic tumor, total RNA sequencing analysis was performed on the tumor tissues. Compared to the original/129 group, the adapted/129 and adapted/B6 groups exhibited many DEGs. Meanwhile, a relatively small number of DEGs were detected between the adapted/129 and adapted/B6 groups (**Figure 3A**).

Gene sets involved in oncogenic signalling, including *Raf*, V*egf*-*a*, and *Akt*, were highly enriched in the adapted/129 and adapted/B6 groups compared with those in the original/129 group (**Figures 3B, C**), in a similar vein, the activities of gene sets in tumor suppressor signalings, such as *Pten* and *P53*, were attenuated (**Figures 3B, D**). Furthermore, gene sets associated with epithelial and mesenchymal stem cell proliferation and cell migration were significantly enriched in the adapted/129 and adapted/B6 groups compared with those in the original/129 group (**Figures 3E, F**). Hence, the oncogenic properties in the adapted cells were more enhanced than the original cells, resulting in more aggressive tumor features.

### Molecular characteristics of the immune microenvironment in adapted TBP3743 cell-derived orthotopic tumors

**Figure 4:**
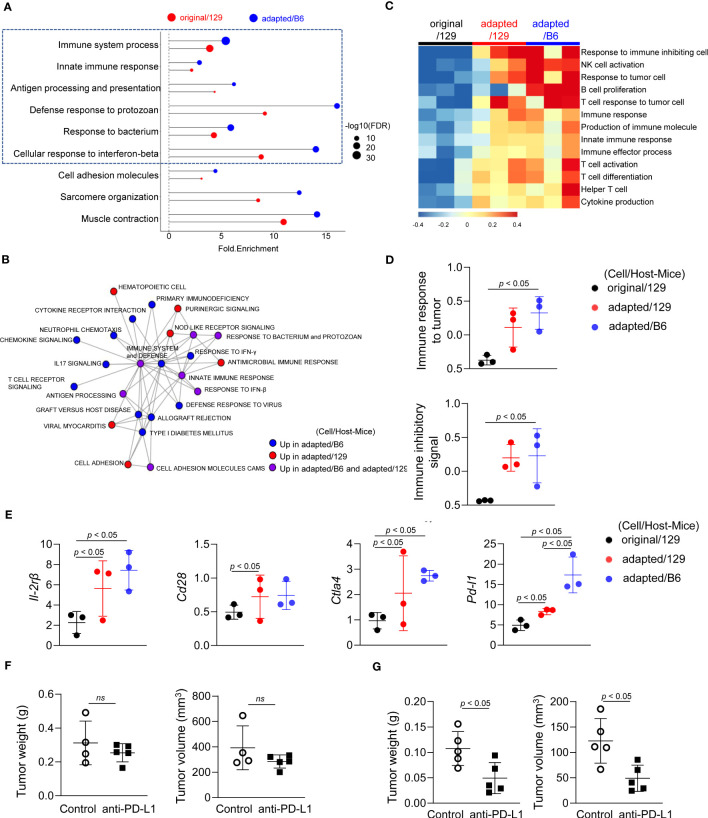
Molecular characteristics of the immune microenvironment in adapted TBP3743 cell-derived orthotopic tumors. B6129SF1 mice were implanted with 10^5^ cells of each cell line (TBP3743-original and -adapted cells) and C57BL/6 mice were implanted with 10^5^ cells of TBP3743-adapted. After 14 days post-implantation, tumors were harvested. Total RNA was harvested from whole tumor lysates and sequenced. **(A)** Classification of gene set enrichment analysis and **(B)** the networking of enriched pathways of immune category. **(C)** Heatmap of changes in immune response signalling pathways and **(D)** change of immune response to tumor and change of immune inhibitory signal. **(E)** Alteration of immune stimulatory related marker and immune inhibitory related marker. **(F)** B6129SF1 mice were implanted with 10^5^ cells of TBP3743-original cells. After 10 days post-implantation, tumors were harvested. Anti-PD-L1 (0.2 mg/kg) was intraperitoneally injected from 5 days after tumor cell injection. Quantification of tumor volume and weight. **(G)** C57BL/6 mice were implanted with 10^5^ cells of TBP3743-adapted cells. After 10 days post-implantation, tumors were harvested. Anti-PD-L1 (0.2 mg/kg) was intraperitoneally injected from 5 days after tumor cell injection. Quantification of tumor volume and weight. 129, B6129SF1; B6, C57BL/6. All data are expressed as mean ± SD.

We next analysed RNA sequencing data to compare the tumor microenvironments among those groups. Notably, immune-related pathways were over-represented in the up-regulated DEGs of the adapted/129 and adapted/B6 groups compared with the original/129 group (**Figure 4A**). Network analysis of the over-represented gene sets further revealed that immune-related pathways were closely organized according to their mutual overlap (**Figure 4B**). Specifically, heatmap analysis demonstrated that the gene sets related to adaptive (T and B cells) and innate (NK T cells and cytokine production) immune responses were upregulated in the adapted/129 or adapted/B6 group compared with the original/129 group (**Figure 4C**). That is, immune response to tumor cells and immune inhibitory signalling pathways were enriched in the adapted/B6 group compared to the original/129 group (**Figure 4D**). Among the immune checkpoint related genes, both immune stimulatory (*Il2rb* and *Cd2*8) and inhibitory (*Ctla4* and *Pdl1*) genes were significantly up-regulated in the adapted/129 or adapted/B6 groups compared with the original/129 group (**Figure 4E**). *In vitro* RT-PCR analysis revealed that inhibitory immune checkpoint genes were up-regulated in the adapted cells compared to the original TBP3743 cells (**Supplementary Table 3**), which were consistent with *in vivo* analysis, while stimulatory immune checkpoint genes were down-regulated in the adapted cells compared with the original TBP3743 cells (**Supplementary Table 3**). Collectively, the immune responses were activated in the adapted/129 and adapted/B6 groups compared with the original/129 group. In the comparison between the adapted/129 and adapted/B6 groups, while it could not be statistically assessed, the fold enrichments were higher in the adapted/B6 group (**Figure 4A**). More over-represented gene sets from the adapted/B6 participated in the network compared with the adapted/129 group (**Figure 4B**). Moreover, the expression of *Pdl1* was considerably higher in the adapted/B6 group than adapted/129 group. These results suggest that the anti-tumor immune responses might be more enhanced in the adapted/B6 group compared with the adapted/129 group.

To validate the distinctive immune characteristics observed in the adapted TBP3743 cell-derived orthotopic tumors, particularly with regard to tumor-T cell immune interactions, additional *in vivo* experiments were conducted under two immune-modifying conditions. Firstly, athymic nude mice, lacking T cell populations including CD4^+^ and CD8^+^ T cells, were utilized to investigate the impact of tumor-T cell interaction on tumor growth. Surprisingly, we observed comparable tumor volumes and weights between the original and adapted cell groups over the 9-day period following tumor cell injection (**Supplementary Figure 5**). These findings indicate the pivotal role of T cells in the tumorigenesis of the adapted model.

To further investigate the interplay between tumor cells and T cells in the adapted model microenvironment, we proceeded with a comparison of the anti-tumor efficacy of an anti-PD-L1 antibody between the original/129 group and the adapted/B6 group. Consistent with a previous study (20), treatment with the anti-PD-L1 antibody alone did not demonstrate a significant treatment response in the original model (**Figure 4F**). However, in the adapted model, both tumor volume and weight were significantly reduced by 60% and 55%, respectively (**Figure 4G**). This finding aligns with the gene expression data presented in **Figure 4E**, which reveals an upregulation of immune checkpoint modifying genes in the adapted model.

### Immune cell profiling of the orthotopic tumor microenvironment

**Figure 5:**
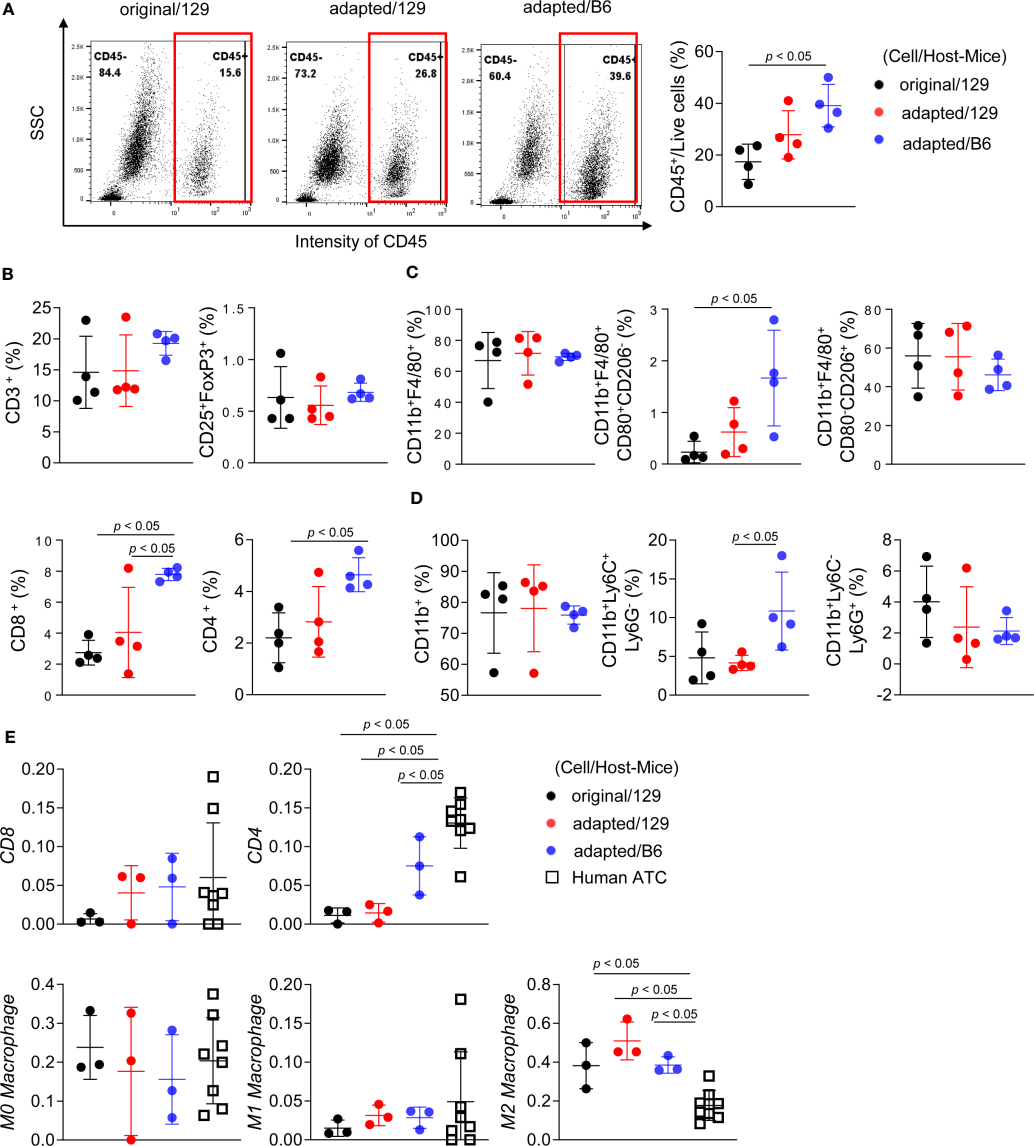
Immune cell profiling of the orthotopic tumor microenvironment. B6129SF1 mice were implanted with 10^5^ cells of each cell line (TBP3743-original and -adapted cells) and C57BL/6 mice were implanted with 10^5^ cells of TBP3743-adapted. After 14 days post-implantation, tumors were harvested. **(A-D)** Immune cell fractions were analysed by flow cytometry analysis using whole tumor lysates. **(A)** Representative plots and quantified graphs of CD45^+^ cells in each tumors. Subpopulation of **(B)** T-lymphocytes (CD3^+^ pan T; CD8^+^ cytotoxic T; CD4^+^ helper T; and CD25^+^FoxP3^+^ regulatory T), **(C)** macrophages (CD11b^+^F4/80^+^ pan macrophage; CD11b^+^F4/80^+^CD80^+^CD206^-^ M1-macrophage; and CD11b^+^F4/80^+^CD80^-^CD206^+^ M2-macrophage), and **(D)** myeloid cells (CD11b+ pan myeloid; CD11b^+^Ly6C^+^Ly6G^-^ M-MDSC; and CD11b^+^Ly6C^-^Ly6G^+^ PMC-MDSC). **(E)** RNA sequencing data of 8 human ATC samples were collected from (8) and deconvolution analysis were performed for comparing immune cell fractions between mice tumors from each group and human ATCs. M-MDSC, mononuclear myeloid-derived suppressor cells; PMN-MDSC, polymorphonuclear myeloid-derived suppressor cells; 129, B6129SF1; B6, C57BL/6. All data are expressed as mean ± SD.

Flow cytometric analysis was additionally performed to characterize immune microenvironment among groups. The CD45^+^ immune cell fraction was significantly greater in the adapted/B6 group compared with the original/129 group (39.1 ± 7.1% vs 17.4 ± 5.9%, *P*< 0.05; **Figure 5A**). The fractions of CD45^+^CD8^+^ cytotoxic T cells and CD45^+^CD4^+^ helper T cells were higher in the adapted/B6 group compared with the original/129 group, while CD45^+^CD3^+^ T cell fraction did not differ among groups (**Figure 5B**). The immune activating M1-macrophage (CD45^+^CD11b^+^F4/80^+^CD80^+^CD206^-^) fraction was greater in the adapted/B6 group compared with the original/129 group (3.5 ± 2.1% vs 0.3 ± 0.3%, *P*< 0.05; **Figure 5C**), while those of total macrophage (CD45^+^CD11b^+^F4/80^+^) and the immune suppressive M2 macrophages (CD45^+^CD11b^+^F4/80^+^CD80^-^CD206^+^) did not differ (**Figure 5C**). The immune suppressive monocytic myeloid-derived suppressor cell (M-MDSC) (CD45^+^CD11b^+^Ly6C^+^Ly6G^-^) fraction was bigger in the adapted/B6 group than the adapted/129 group (10.6 ± 4.7% vs 4.1 ± 0.9%, *P*< 0.05; **Figure 5D**). Flow cytometric subset analysis for T cell and macrophage in the original/129, the adapted/B6, and the SC6/B6 also supported this notion. Those immune cell changes observed between the original and adapted model did not differ between the adapted/B6 and SC6/B6 (**Supplementary Figure 6**). Collectively, both activating and suppressive immune responses were up-regulated in the adapted/B6 group compared with the original/129 group. However, these differences were not observed between the original/129 and adapted/129 groups.

In addition to the increased immune infiltrate, especially characterized by increased T cell, immune escape cause by losing major histocompatibility (MHC) class I molecule can be another possible immune response in the process of the adapted cell tumorigenesis (24). Thus, we further analysed the expression of MHC class I molecules. *In vitro* mRNA expression of MHC class I gene was not lost, rather significantly upregulated in both SC3 and SC6 cells compared with the original cell (**Supplementary Figure 7A**). Additionally, flow cytometric analysis showed that under interferon-γ stimulation, the functional expression of MHC class I in SC3 cells was comparable to that of original cells, while SC6 cells showed a decrease in interferon-γ-mediated upregulation of MHC class I expression (**Supplementary Figure 7B**). These results suggest that immune escape through functional loss of MHC class I molecules may contribute to tumorigenesis in adapted cells, particularly in the later phase (represented by SC6 cells) rather than the early phase (represented by SC3 cells).

A bulk mRNA sequencing-based deconvolution analysis was performed to compare the immune profiles of murine tumor models to those of human ATC harbouring BRAF mutation. **Supplementary Table 4** showed baseline characteristics of them (n=8). In the comparisons of fractions in CD4^+^ T cell, CD8^+^ T cell, and macrophages, human ATC showed higher CD4^+^ T cell fractions than the murine tumors (**Figure 5E**). Given that heavy infiltration of M2-like macrophage is the characteristics of human ATC (3), M2-like macrophage fraction of human ATC was 16.5% (12.6%-21.1%). Those of the murine tumors were 38.4% (32.3%-44.2%) in the original/129 group, 45.4% (45.3-53.8%) in the adapted/129 group, and 36.3% (36.1%-39.9%) in the adapted/B6 group, which were all higher than that of human ATC.

### Effects of a BRAF inhibitor on adapted TBP3743 cell-derived orthotopic tumors

**Figure 6:**
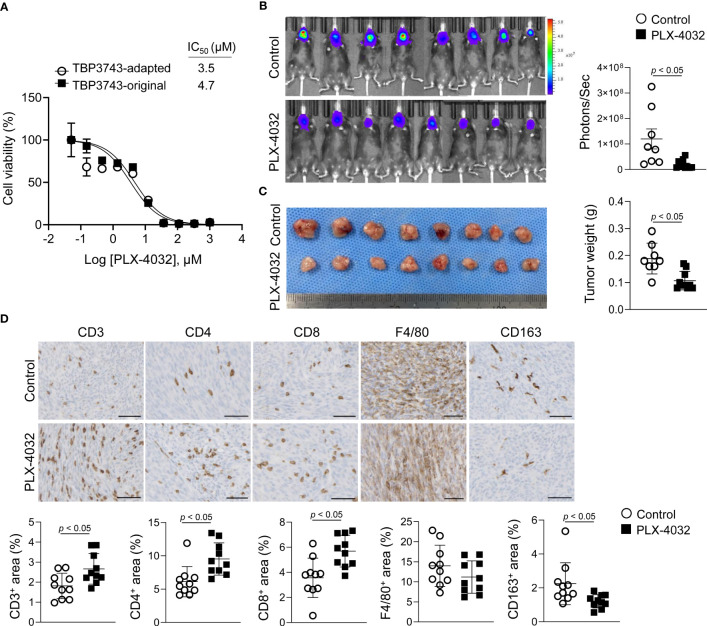
Effects of *BRAF* ^V600E^ inhibitor on TBP3743 cells. **(A)** Effect of PLX-4032 on TBP3743-original and -adapted cells were measured by cell viability assay. **(B-D)** TBP3743-adapted cells (1 × 10^5^/10µL PBS) were injected into the murine thyroid via orthotopic injection, and mice were daily treated with 10 mg/kg of PLX-4032. At 2 weeks after injection, tumors were harvested. **(B)** bioluminescence imaging and **(C)** representative image of tumors, and quantification of tumor weight. **(D)** Representative images of IHC for T cell (CD3), helper T cell (CD4), cytotoxic T cell (CD8), macrophage (F4/80), and M2-macrophage (CD163) in each group tumors. scale bars: 50 µm. All data are expressed as mean ± SD.

Finally, drug responses to PLX-4032, a selective BRAF^V600E^ kinase inhibitor, were explored *in vitro* and *in vivo*. *In vitro*, the inhibitory effects of PLX-4032 on cell viability were similar between the original (IC_50,_ 4.7 µM) and adapted (IC_50,_ 3.5 µM) cells (**Figure 6A**). However, PLX-4032 treatment in the adapted/B6 group significantly reduced tumor burden by 84%, as determined by bioluminescence imaging, and tumor weight by 43% compared with the untreated group (**Figures 6B, C**).

Considering that a previous study reported immune modulatory effects of BRAF kinase inhibitor (20), the T cell and macrophage densities within the tumor microenvironment were assessed by IHC. Consistent with the previous study (20), the CD3^+^, CD4^+^, and CD8^+^ T cell proportions were increased in the PLX-4032-treated group compared to the untreated group (**Figure 6D**). Moreover, the F4/80^+^ macrophage proportion exhibited no difference, while that of CD163^+^ M2-macrophages was decreased (**Figure 6D**). Collectively, the adapted TBP3743 cells responded to PLX-4032 appropriately, and the adapted/B6 model proved applicable for evaluation of the anti-tumor efficacy and immune modulatory effects of drugs.

## Discussion

In the present study, we adapted the B6129SF1 mouse-derived ATC cell line, TBP3743, harbouring *Braf^V600E^* mutation and *Trp53* deletion (19), into C57BL/6 inbred mice and established a novel orthotopic tumor model of ATC. Although the adapted TBP3743 cells were de-dedifferentiated, they exhibited similar gross morphology, cell viability, and migration/invasion potential as the original TBP3743 cells *in vitro*. Moreover, our novel orthotopic tumor model in C57BL/6 mice showed similar histological features to human ATC with high oncogenic properties and activated anti-tumor immune responses. Indeed, the anti-tumor effects and immune modulatory effects of a BRAF inhibitor were observed in the model.

The adapted cells represented de-differentiated clones. During *in vivo* serial passaging, PAX8 expression was completely lost, while the expressions of TTF-1 and TSH-R were low but preserved. In contrast to the typical pattern observed in human ATC, where PAX8 is generally preserved (25, 26) and TTF-1 and TSH-R are lost earlier in the de-differentiation process (27), the TBP3743 cell line used in our study exhibited a different order of gene deletion. The adapted cells showed a reduction in mRNA expression of *TTF-1* and *TSH-R* compared to the original cells, but preservation of protein expressions of TTF-1, while the protein levels of TSH-R were negligible. Additionally, the recently described ATC marker gene, IGF2BP1 (23), was preserved in both the original and adapted cells. These findings indicate that the adapted ATC cells were still undergoing the de-differentiation process observed in human thyroid cancer.

The histologic features of the original/129, adapted/129, and adapted/B6 cells were consistent with human sarcomatoid ATC, characterized by spindle sarcoma-like tumor cells with pleomorphic nuclei and a high mitotic count. The complete loss of PAX8 in the adapted cells can be explained by previous reports indicating low positivity of PAX8 in human sarcomatoid ATC (less than 50%) (25, 26). Therefore, the adapted cells and the adapted/B6 model, paired with the original cells and original/129 model, provide a valuable tool for investigating the pathophysiology of the de-differentiation process in ATC.

The present study focused on analysing the molecular characteristics of ATC tumors using RNA sequencing data. *In vitro* analysis revealed that the gross cancer cell behaviours, including cell proliferation rates, with or without BRAF inhibitors, or migration/3D invasion potentials, were similar between the original and adapted cells. However, the rate of *in vivo* tumor growth and the Ki-67 index was significantly higher in B6129SF1 mice injected with the adapted TBP3743 cells compared to those injected with original TBP3743 cells. Molecular analyses supported these phenotypic differences demonstrating that gene sets associated with driver mutation pathways, such as RAF upregulation and PTEN and P53 down-regulation, as well as other oncogenic pathways, were enhanced in the tumors derived from adapted TBP3743 cells compared to original cells in B6129SF1 mice. These findings highlight the marked differences in cancer cell behaviour within *in vivo* and *in vitro* systems; thus, *in vitro* results must be interpreted carefully.

The evaluation of MHC molecule expression provides further insight into the adaptation mechanism studied here. In general, the selected clones exhibited loss of MHC genes, which has the potential to lead to immune evasion (28). Interestingly, the original cells and SC3 clone, representing an early adapted cell, retained their antigen-presentation functions by expressing MHC class I molecules. However, the SC6 clone exhibited significant loss of functional MHC class I under interferon-γ stimulation, highlighting the critical role of immune evasion in the later stages of the adapted model, while not observed in the early adaptation model. Therefore, careful selection and optimization of subclone passages are necessary for further study.

Interestingly, the tumor growth rate differed between murine hosts (i.e., B6129SF1 391 versus C57BL/6), even following the implantation of the same adapted TBP3743 cells. The adapted TBP3743 cells enhanced immune responses to tumors in all mice; however, the effect was stronger in C57BL/6 than in B6129SF1 mice. Not only were the immune stimulatory responses, including cytotoxic T cell or M1 macrophage proportions, enhanced in C57BL/6 mice compared to B6129SF1 mice, but also the immune suppressive responses, such as the M-MDSC proportion and *Pdl1* expression. This homeostasis of immune responses might account for the overall reduced tumor growth in C57BL/6 mice compared with B6129SF1 mice. Indeed, these findings highlight the importance of the interactions between the tumor microenvironment and cancer cells. This was further demonstrated *in vitro* by the downregulated expression of immune checkpoint genes in the adapted TBP3743 cells *in vitro*, which became upregulated following *in vivo* generation of tumors within C57BL/6 mice. Hence, cancer cells exhibit dynamic molecular responses to the host immune responses.

ATC stands out among various solid cancers as a highly immunogenic tumor characterized by dense infiltration of tumor-associated macrophages (TAMs) (3). Additionally, recent single-cell transcriptomics data have demonstrated that the tumor immune microenvironment undergoes significant reprogramming during ATC progression. This reprogramming is initiated by the substantial infiltration of immune cells, including macrophages and T cells, during the anaplastic transformation (28). Hence, understanding cancer-immune cell interactions is crucial in ATC research (29, 30). Our deconvolution analysis of total RNA sequencing data confirms the abundant presence of CD4^+^ and CD8^+^ T cells in human ATC samples, consistent with previous studies (31). Notably, the adapted/B6 model closely recapitulates the degree of tumor-infiltrating T cells observed in human ATC samples compared to other murine tumor groups. With the development of various T cell-targeting anti-tumor drugs over the past decade (32–34), some of which have demonstrated success in clinical settings (35–37), our model provides an improved platform for the ongoing advancement of immune-modulating therapeutics for ATC. Specifically, it enables more sensitive verification of the anti-tumor and immune-modulatory effects of candidate drugs in a model that closely mirrors the immune response observed in human ATC. Furthermore, the adapted model’s median survival which is approximately 14 days offers an extended timeframe to assess drug response. Indeed, the adapted model demonstrated a notable response to anti-PD-L1 therapeutics, whereas the original model exhibited limited efficacy, consistent with previous findings (20).

Overall, the pros and cons of the adapted model is summarized as follows. The adapted model is the representative model of human ATC: The adapted cells are de-differentiated, possess molecular characteristics of human ATC. *In vivo* tumor with the adapted cells in C57BL/6 results in heavy infiltration of immune cells, which possibly resembles distinct tumor-host immune interaction of human ATC. The tumor growth rate and median survival is appropriate to investigate responses of anti-tumor drugs. Despite our interpretation that the adapted model represents the characteristics of de-differentiated clones and enhanced immune response similar to “hot tumors” in human ATCs, it is possible that the observed enhanced immunity is partially due to simple allogenic rejection. Additionally, while we claimed that the prolonged survival in the adapted model is more suitable for drug development, it may raise concerns that this prolongation conflicts with the features of human ATC. However, it should be noted that the median survival of our model (16.5 days) is significantly shorter than the orthotopic mouse model using the 8505c human ATC cell-line, which showed steady tumor growth over 35 days without any deaths (38).

In conclusion, a novel orthotopic tumor model of ATC has been established by successful cell adaptation via *in vivo* passage in inbred C57BL/6 mice. This new syngenic ATC mouse model will advance the current understanding of the tumor microenvironment, particularly from an immunological context, and will prove effective as a preclinical platform for ATC drug discovery.

## Data availability statement

The data presented in the study are deposited in the GEO repository, accession number GSE234282. The other data used in this study are available from the authors upon request.

## Ethics statement

The animal study was reviewed and approved by The Institutional Animal Care and Use Committee approved the protocols for the animal study (No. SNU-190214-1-3), and all animals were maintained in accordance with the Guidelines for the Care and Use of Laboratory Animals of the Institute of Laboratory Animal Resources, Seoul National University.

## Author contributions

ZX and HS performed all *in vivo* experiments and generated the data. HS and SH performed *in vitro* experiments and generated the data. YK analysed mRNA sequencing data and generated the figures. JKW performed histologic analysis and provided important insights. ZX, HS, YK, and SC wrote and edited the manuscript. S-jK, YP, and SP reviewed and edited the manuscript. SC and KL conceptualized the study and supervised the project. KL was responsible for funding acquisition for this study. All authors contributed to the article and approved the submitted version.
