# Supplementary material for: Modeling the tumor microenvironment of anaplastic thyroid cancer: an orthotopic tumor model in C57BL/6 mice
Source: Front Immunol. 2023 Jul 21;14:1187388. doi: 10.3389/fimmu.2023.1187388 (PMC10403231; doi:10.3389/fimmu.2023.1187388)
Supplement: Supplementary file 1 [file DataSheet_1.docx]

**Material & Methods for Supplementary data

Western blot analysis**

For Western blot analysis, cell lysates were collected using the RIPA buffer (Thermo). The following antibodies were used: anti-pErk (1:1000, 9101S; Cell Signaling), anti-Erk (1:1000, 9102S; Cell Signaling), anti-pAkt (1:1000, 9271S; Cell Signaling), anti-Akt (1:1000, 9272S; Cell Signaling), anti-E-cadherin (1:1000, #3195; Cell Signaling), anti-N-cadherin (1:1000, #14215; Cell Signaling), anti-Vimentin (1:1000, #5741; Cell Signaling), anti-PAX8 (1:1000, MA1-117; Invitrogen), anti-TTF-1 (1:1000, Santa cruz, sc-53136), anti-TSHR (1:1000, Santa cruz, sc-53542), anti-β-actin (1:1000, Sigma-Aldrich, A5441), and anti-GAPDH (1:1000, #2118; Cell Signaling). The results are presented as the average of three independent experiments.

**Flow cytometry for MHC class I**

Both Original and adapted (SC3 and SC6 clones) TBP3743 cells were plated in 6-well plates at a density of 3 × 10^5^ cells/wellRecombinant mouse interferon-γ protein (50 ng/ml, R&D Systems, Minneapolis, MN) was added and the cells were treated for 24 hours. After treatment, the cells were detached using Trypsin-EDTA (0.25%) (Gibco, 252200056) and suspended in PBS supplemented with 2% FBS. Subsequently, the cells were stained with FITC-conjugated anti-MHC class I antibody (1:50, Abcam, Cambridge, MA, ab95572). Flow cytometry was performed using the MACSQuant® Analyzer 16 (Miltenyi Biotec B.V. & Co. KG, Bergisch Gladbach, Germany), and the data were analyzed using FlowJo software (BD Biosciences, OR, USA).
