# Supplementary figures and images for: Modeling the tumor microenvironment of anaplastic thyroid cancer: an orthotopic tumor model in C57BL/6 mice
Source: Front Immunol. 2023 Jul 21;14:1187388. doi: 10.3389/fimmu.2023.1187388 (PMC10403231; doi:10.3389/fimmu.2023.1187388)

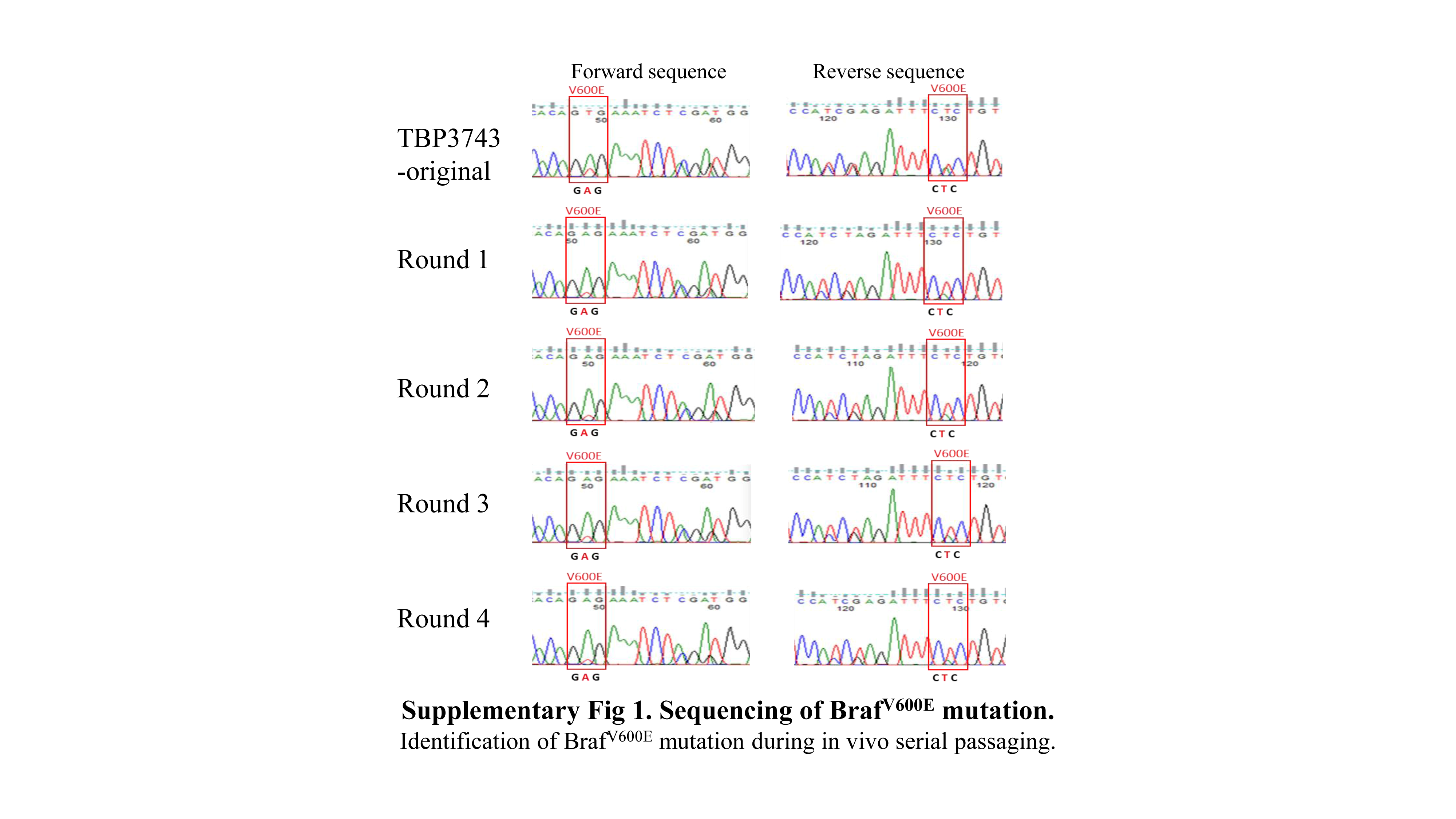

Supplement: Supplementary file 2 [file Image_1.tif]

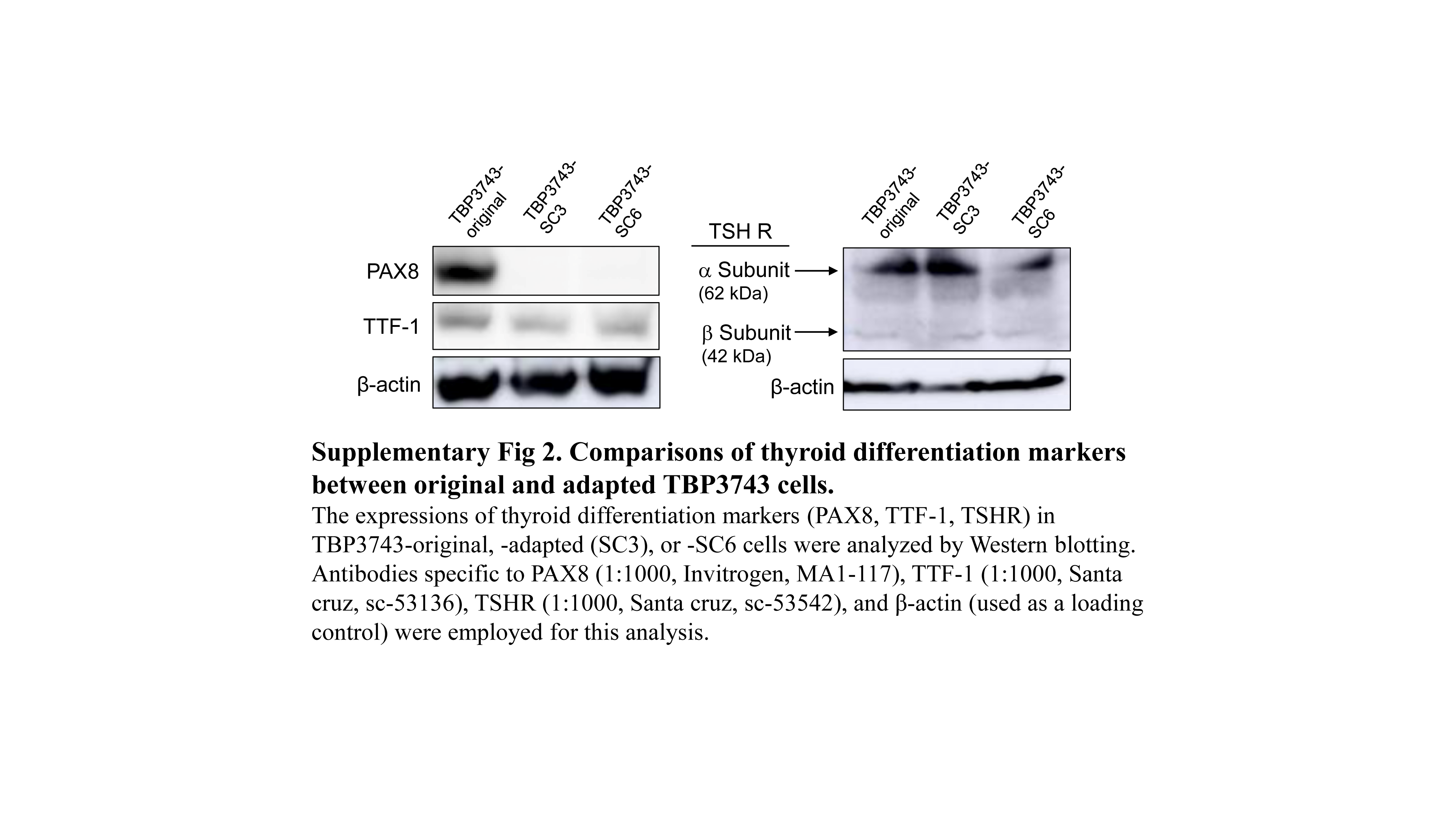

Supplement: Supplementary file 3 [file Image_2.tif]

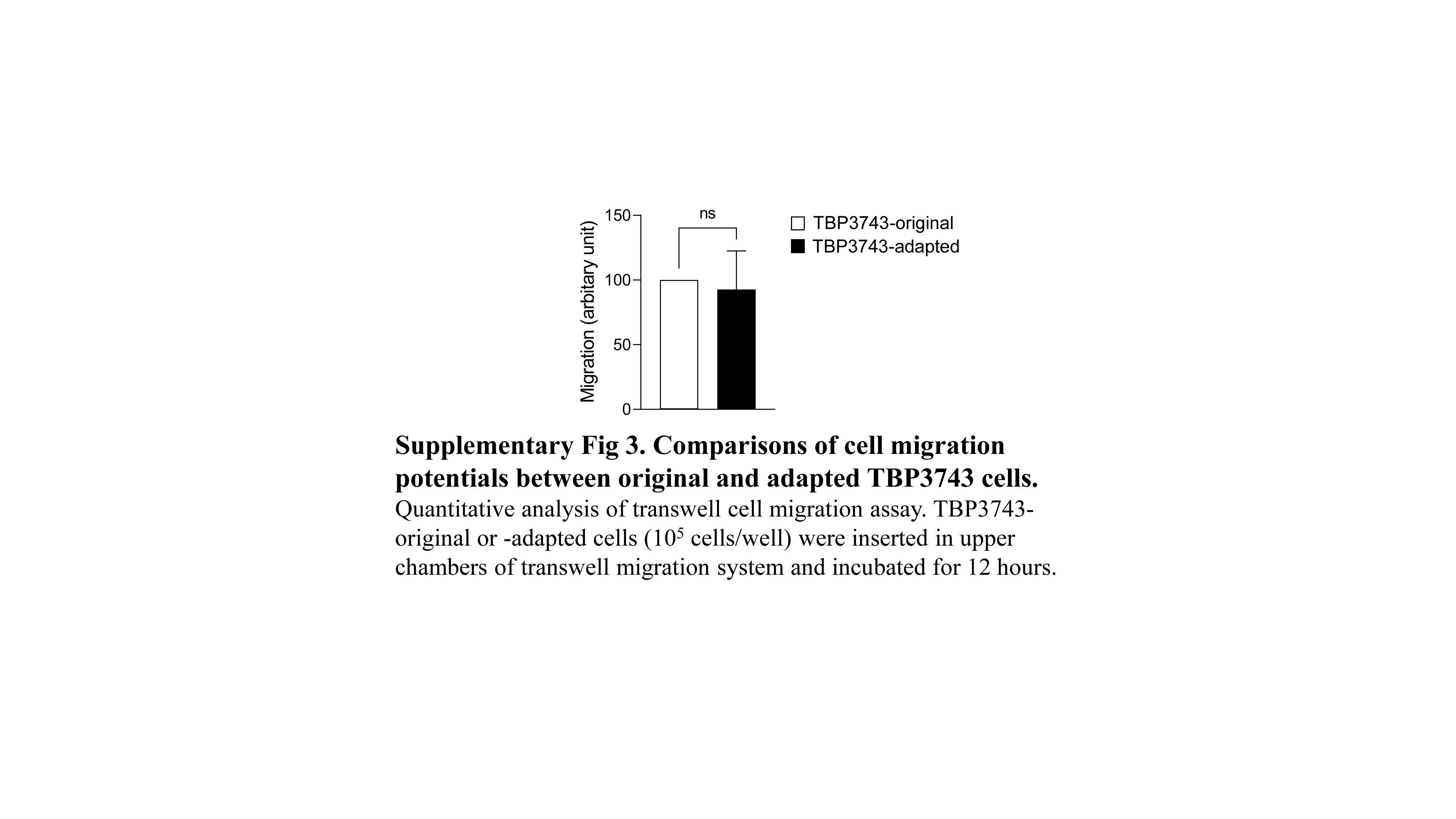

Supplement: Supplementary file 4 [file Image_3.tif]

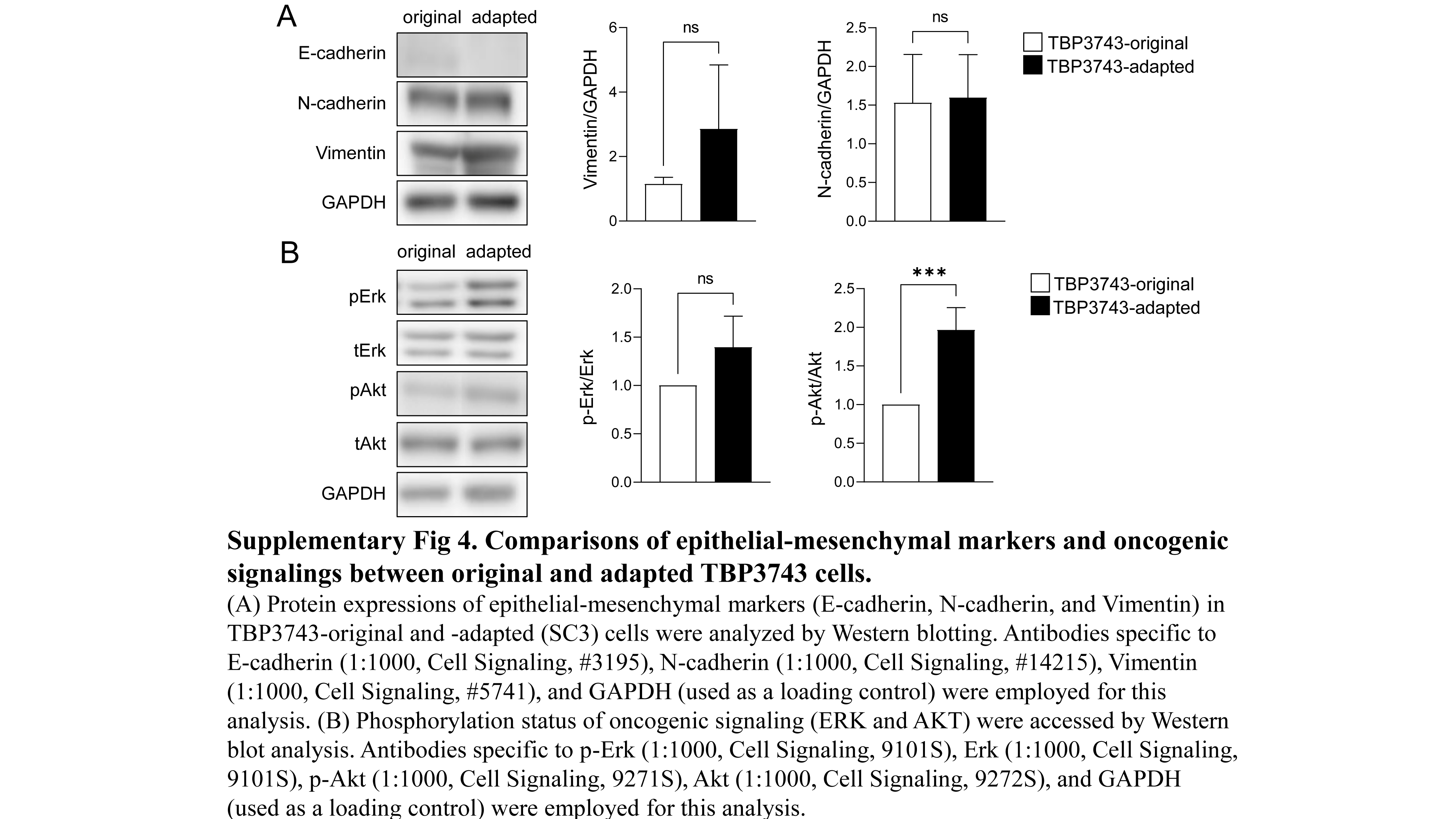

Supplement: Supplementary file 5 [file Image_4.tif]

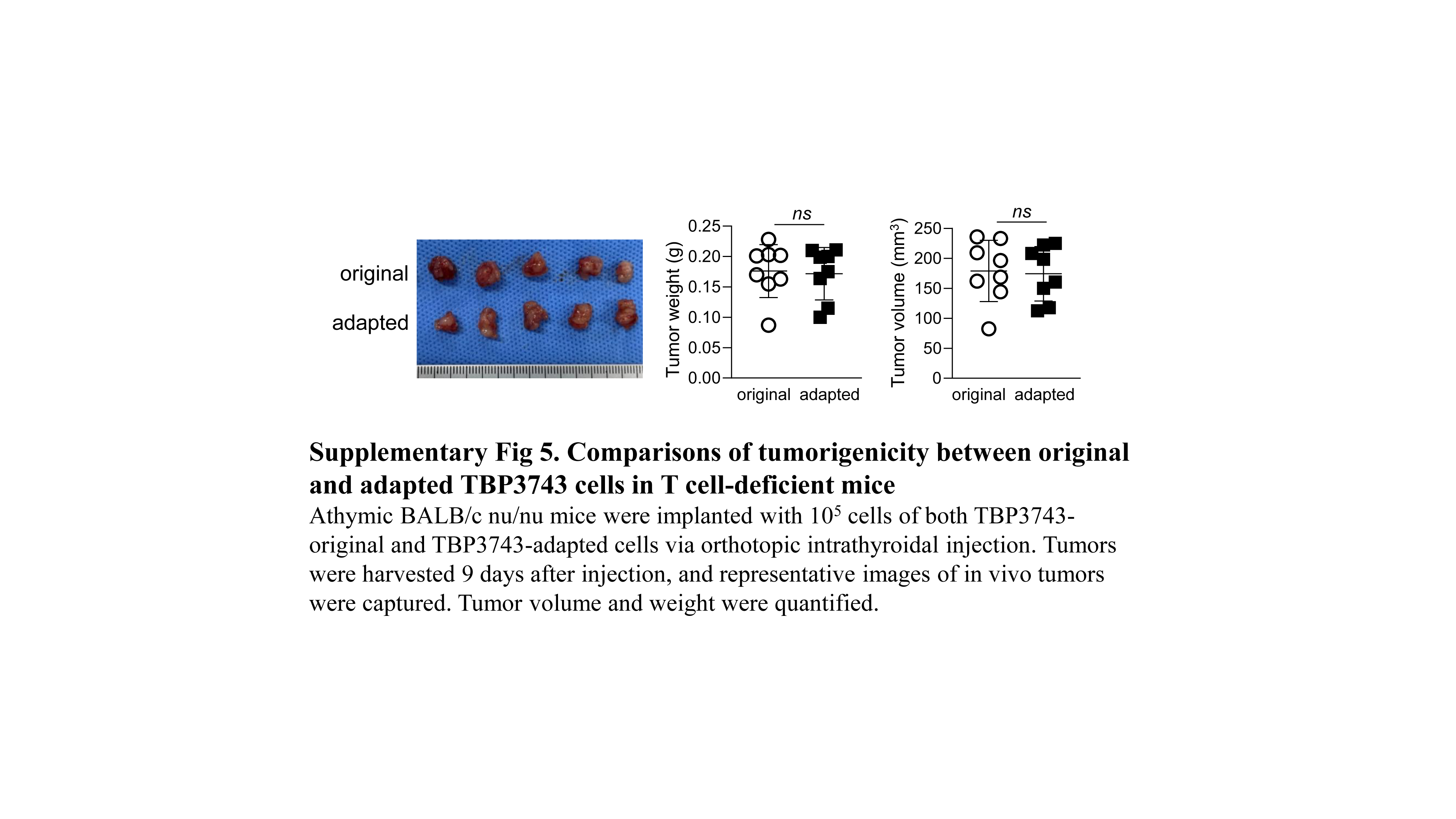

Supplement: Supplementary file 6 [file Image_5.tif]

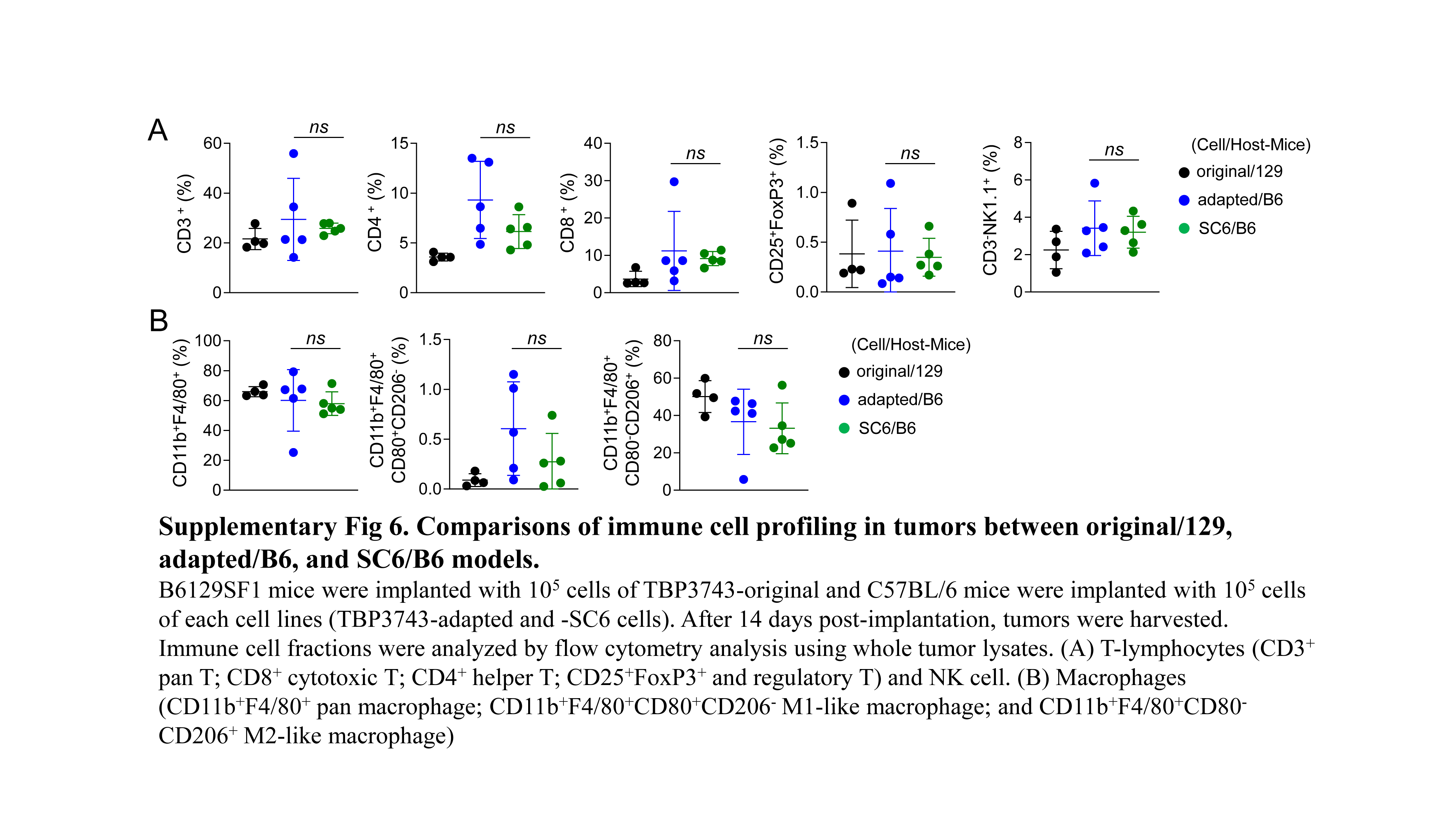

Supplement: Supplementary file 7 [file Image_6.tif]

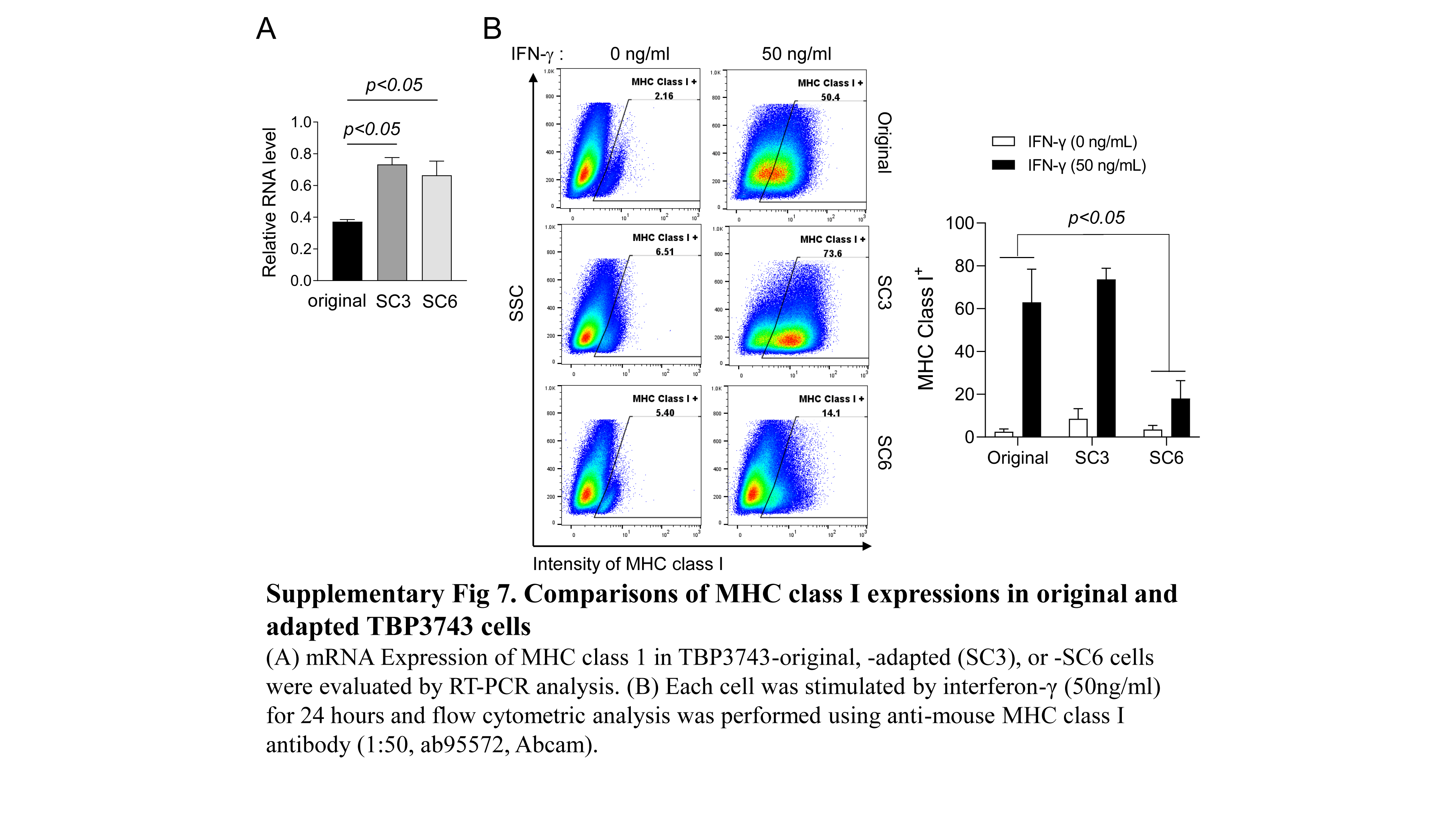

Supplement: Supplementary file 8 [file Image_7.tif]

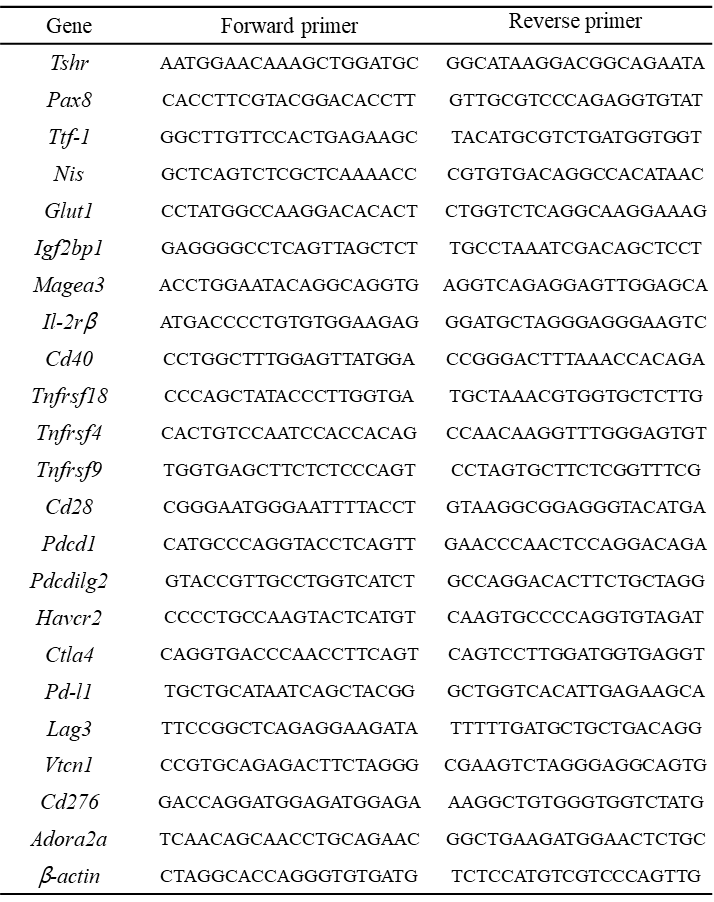


Supplementary Table 1. Primer sequences

Supplement: Supplementary file 9 [file Table_1.docx]

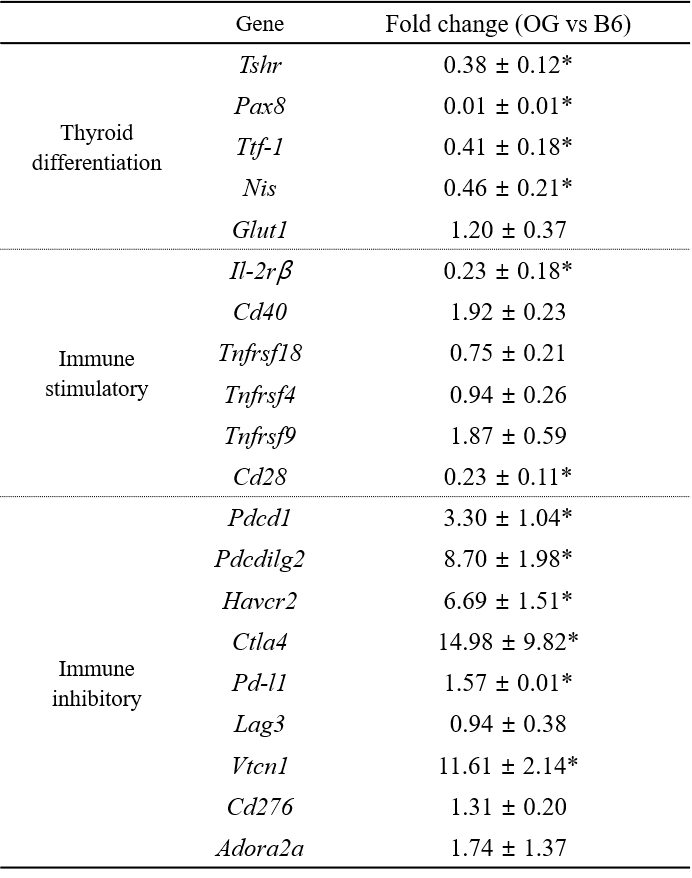


Supplementary Table 3. List of genes for RT-qPCR

**P* < 0.05

Supplement: Supplementary file 11 [file Table_3.docx]
