# Supplementary material for: Modeling the tumor microenvironment of anaplastic thyroid cancer: an orthotopic tumor model in C57BL/6 mice
Source: Front Immunol. 2023 Jul 21;14:1187388. doi: 10.3389/fimmu.2023.1187388 (PMC10403231; doi:10.3389/fimmu.2023.1187388)
Supplement: Supplementary file 10 [file Table_2.docx]

Supplementary Table 2. The number of measurable tumors according to the round of *in vivo* passage

|  | Days after tumor cell injection | | | |
| --- | --- | --- | --- | --- |
|  | 7 | 14 | 21 | 28 |
| Round 1 | -/5 | -/5 | -/5 | 1/5 (20%) |
| Round 2 | -/5 | -/5 | 1/5 (20%) | 3/5 (60%) |
| Round 3 | 2/5 (40%) | 4/5 (80%) | 5/5 (100%) | 5/5 (100%) |
| Round 4 | 1/5 (20%) | 5/5 (100%) | 5/5 (100%) | 5/5 (100%) |
| Round 6 | 5/5 (100%) | 5/5 (100%) | 5/5 (100%) | 5/5 (100%) |

Measurable tumors were defined as a tumor volume over 400 mm^3^.
