# Supplementary material for: Modeling the tumor microenvironment of anaplastic thyroid cancer: an orthotopic tumor model in C57BL/6 mice
Source: Front Immunol. 2023 Jul 21;14:1187388. doi: 10.3389/fimmu.2023.1187388 (PMC10403231; doi:10.3389/fimmu.2023.1187388)
Supplement: Supplementary file 12 [file Table_4.docx]

Supplementary Table 4. Clinical characteristics of human ATC

|  | Human ATC |
| --- | --- |
| Number | 8 |
| Age, years | 71 ± 10.6 |
| Female, n (%) | 7 (87.5%) |
| BRAF^V600E^, n (%) | 8 (100%) |
| TERT promoter mutation, n (%) | 8 (100%) |
| Tumor size, cm | 4.5 ± 2.1 |
| Extrathyroidal extension, n (%) | 8 (100%) |
| Lymph node metastasis, n (%) | 8 (100%) |
| Distant metastasis, n (%) | 6 (75.0%) |
| Disease specific death, n (%) | 7 (87.5%) |
